# Hospitals and Their Detractors

**Published:** 1897-12-04

**Authors:** 


### HOSPITALS AND THEIR DETRACTORS.

It is to be hoped that some metropolitan magistrates are more careful as to facts in deciding the cases that come before them than they sometimes seem to be in regard to the numerous side issues upon which they so often presume to express irresponsible and absurd opinions. As an example of the harm done by the free and easy manner in which some magistrates, who like to air their opinions on things at large, scatter calumnies on innocent persons and institutions, we reprint the following correspondence from the *Times* :—

### MR. CURTIS BENNETT AND UNIVERSITY COLLEGE HOSPITAL.

TO THE EDITOR OF THE TIMES.

Sir,—I am requested by the Hospital Committee of University College Hospital to ask you to publish the enclosed letter, which was sent by me to Mr. H. Curtis Bennett, metropolitan police magistrate for the Marylebone district, on November 15th. Mr. Bennett has neither acknowledged nor replied to this communication, and it has therefore been forwarded to the Home Office.

We can ill afford to submit patiently to blame for that which we have not committed, especially at a time when our support from the public is so inadequate that the temporary closure of fifty beds has become an absolute necessity.

There are, moreover, no means of denying such irresponsible accusations from the Bench without appealing to the public Press.—I am, Sir, yours faithfully,

Rickman J. Godlee.

19, Wimpole Street, W., November 24th.

To H. Curtis Bennett, Esq , Metropolitan Police Magistrate for the Marylebone District.

November 15th, 1897.

Dear Sir,—As Dean of the Medical Faculty of University College, it has been brought to my notice that in the police reports of the *Times* newspaper of November 13th the following appears :—

"Mr. Curtis Bennett, addressing the witness" (Mr. Alfred Trotter), "said that when the prisoner" (James Harris) "was brought to this Court he was not in a fit condition to bear the strain. This was the second time this kind of thing had occurred in connection with the University College Hospital within the last two months." And again: "Mr. Curtis Bennett observed that the man was to be brought before a Court of Justice, and his condition was such that he was absolutely unfit to go through the ordeal. If such a thing happened again he should make a report of the circumstances to the Home Office."

You must be aware that statements of this kind are calculated to do harm to the institution about which they are made, and, unless they are warranted by facts, are not justifiable. I desire to point out to you (1) that for four days before his discharge from University College Hospital the patient had been sitting up from two to four hours daily, and was in fact convalescent. (2) That the house-surgeon in charge of the case wrote to the divisional police-surgeon stating the full facts of the case, and saying that when they could arrange for the proper medical supervision of the prisoner he might be removed. (3) That next day the police-constable called and asked when he might go. The house-surgeon replied that he had written to the doctor, and when he could arrange for the reception of the prisoner he might be removed. (4) That afterwards Inspector Cox called and asked the house-surgeon if, on the way to Holloway Infirmary, he mi ht take the prisoner to Marylebone Police-court to prove his arrest, and promised only to keep him there ten minutes. The house-surgeon replied that he might be taken from the hospital to the divisional police surgeon in a cab, the surgeon must accompany him to the court, and when the arrest was proved he must be further driven straight to Holloway in a cab, as he was not fit for a prolonged trial at that time. (5) That on the occasion on which you are reported to have made the observations I have quoted he was brought into court, not from University College Hospital, but by the police authorities, presumably from Holloway Gaol.

I have reason to believe that these facts were brought before you by Mr. Trotter, and I should be glad to know whether, in the face of them, you consider your observations, as reported, are justified; and, if not, whether you will see your way to making some public explanation of them.

I should also be glad to know which is the other occasion on which you are reported to have said "this kind of thing had occurred in connection with University College Hospital within the last two months," as, after inquiry at the hospital, I am unable to hear of any such case.

Failing some explanation, it will be my duty, in the interest of University College Hospital and other institutions of the sort, to forward this correspondence to the Home Office for consideration by the authorities.—I am, yours faithfully,

R. J. Godlee.

